# Selecting Habitat to Survive: The Impact of Road Density on Survival in a Large Carnivore

**DOI:** 10.1371/journal.pone.0065493

**Published:** 2013-07-10

**Authors:** Mathieu Basille, Bram Van Moorter, Ivar Herfindal, Jodie Martin, John D. C. Linnell, John Odden, Reidar Andersen, Jean-Michel Gaillard

**Affiliations:** 1 Fort Lauderdale Research and Education Center, University of Florida, Fort Lauderdale, Florida, United States of America; 2 Chaire de recherche industrielle CRSNG-Université Laval en sylviculture et faune, Département de biologie, Université Laval, Québec, Canada; 3 Laboratoire de Biométrie et Biologie Évolutive, UMR 5558, Université de Lyon, Université Lyon 1, CNRS, Villeurbanne, France; 4 Norwegian Institute for Nature Research (NINA), Trondheim, Norway; 5 Centre for Biodiversity Dynamics, Department of Biology, Norwegian University of Science and Technology, Trondheim, Norway; 6 Centre for African Ecology, School of Animal, Plant and Environmental Sciences, University of the Witwatersrand, Wits, South Africa; 7 Norwegian Institute for Nature Research (NINA), Trondheim, Norway; 8 Vitenskapsmuseet, Norwegian University of Science and Technology, Trondheim, Norway; University of Alberta, Canada

## Abstract

Habitat selection studies generally assume that animals select habitat and food resources at multiple scales to maximise their fitness. However, animals sometimes prefer habitats of apparently low quality, especially when considering the costs associated with spatially heterogeneous human disturbance. We used spatial variation in human disturbance, and its consequences on lynx survival, a direct fitness component, to test the Hierarchical Habitat Selection hypothesis from a population of Eurasian lynx *Lynx lynx* in southern Norway. Data from 46 lynx monitored with telemetry indicated that a high proportion of forest strongly reduced the risk of mortality from legal hunting at the home range scale, while increasing road density strongly increased such risk at the finer scale within the home range. We found hierarchical effects of the impact of human disturbance, with a higher road density at a large scale reinforcing its negative impact at a fine scale. Conversely, we demonstrated that lynx shifted their habitat selection to avoid areas with the highest road densities within their home ranges, thus supporting a compensatory mechanism at fine scale enabling lynx to mitigate the impact of large-scale disturbance. Human impact, positively associated with high road accessibility, was thus a stronger driver of lynx space use at a finer scale, with home range characteristics nevertheless constraining habitat selection. Our study demonstrates the truly hierarchical nature of habitat selection, which aims at maximising fitness by selecting against limiting factors at multiple spatial scales, and indicates that scale-specific heterogeneity of the environment is driving individual spatial behaviour, by means of trade-offs across spatial scales.

## Introduction

Habitat selection is generally assumed to be an adaptive behaviour, by which animals choose particular habitat attributes and food resources to maximise their fitness [1]. However, animals do not always correctly assess habitat quality, and a mismatch between the environmental cues they use to select their habitat and actual habitat quality can result in animals sometimes preferring habitats of apparently low quality [2]. Such maladaptive habitat selection [3] often occurs in habitats modified by human activities, or more generally in rapidly changing landscapes [2]. Individual variation in Darwinian fitness is known to occur in relation to habitat features, especially in the presence of spatial heterogeneity in human activities [4]. Habitat characteristics at a large range of scales influence animal performance at virtually all levels of biological organisation, from fine scale characteristics of feeding patches that determine individual energy gain [5] to landscape characteristics that drive population growth [6]. The relationship between habitat selection and fitness should thus also be scale-specific [7], [8] to reflect the hierarchy of factors potentially limiting individual fitness.

In a landmark paper, Rettie and Messier [9] proposed that the hierarchy of habitat selection for a given individual should reflect the hierarchy of factors potentially limiting its fitness. This hypothesis, hereafter named the Hierarchical Habitat Selection (HHS) hypothesis, states that the most limiting factor should drive behaviour at coarser spatial scales, and be less influential at finer spatial scales until the next most limiting factor takes precedence over it. In the context of predator-prey relationships, it has been suggested that species mostly limited by predation should exhibit a strong avoidance of risky areas at large scales, while the search for high quality food should predominate at finer scales [9]. Following the original study by Rettie and Messier on woodland caribou *Rangifer tarandus caribou* [9], the HHS hypothesis has received mixed support from empirical analyses in a large range of animal species. A first set of studies did not reveal any selection against the most limiting factors (being food limitation or predation risk) at large scales in contrast to fine-scale selection (e.g. [10], [11]). Moreover, several studies over a large range of species have demonstrated a consistent selection pattern across scales (e.g. [12]–[15]), leading to the rejection of the HSS hypothesis. On the contrary, some studies provided clear support for the hierarchical nature of habitat selection. For example, migratory elk *Cervus elaphus* strongly reduced their exposure to wolf *Canis lupus* predation at the landscape scale, and preferred areas with intermediate forage digestibility at the fine scale [16]. Interestingly, however, resident elk simultaneously avoided predation risk and selected for maximum forage biomass at fine spatial scales, due to a decoupling between food and risk originating from human activity. Similarly, woodland caribou (in a different area than in [9]) directed their large-scale movements to avoid predation risk, and their fine-scale movements towards foraging areas [17]. Altogether, these studies emphasise the key importance of trade-offs (generally between energy intake and mortality risk) at a given spatial scale [18]. However, to our knowledge, no study, including Rettie and Messier's original study, has assessed habitat selection at different scales with an associated direct component of fitness to define the limiting factors at each spatial scale.

Nowadays, a large proportion of environmental variation is human-caused. Humans impact animal performance either directly, notably through disturbance, hunting or poaching [19], [20], or indirectly by altering habitat [21] or changing food chain equilibrium [22], [23] and climate [24]. Altogether, human disturbance can heavily affect the spatial heterogeneity of resources in the environment, which in turn can markedly impact population dynamics [25], [26], and, thereby, the selective pressure on habitat selection. To reliably assess human impacts on animal fitness, it is crucial to understand how space use affects individual performance by relating habitat use and selection with fitness components over a continuum of spatial scales [4], [27].

In this study, we implemented a formal test of the HHS hypothesis, by relating a direct component of individual fitness, adult mortality, and habitat selection at two spatially nested scales. We investigated the relationship between home range composition (second-order selection from [28]), habitat selection (third-order selection from [28]) and mortality for the Eurasian lynx *Lynx lynx* in southern Norway, and how lynx responded to this relationship. Lynx in southern Norway occupy relatively large home ranges (300–3,000 km^2^ [29], [30]), and thrive in a human-dominated landscape [31]. Lynx are currently managed through a quota hunting system that aims to stabilise their population density and attempts to limit depredation on domestic sheep [32]. As a consequence, mortality of lynx is mostly human-caused, with nearly 90% of mortality events reported in Scandinavian lynx due to legal hunting (43%) and poaching (47%) [33], which makes humans the most limiting factor for lynx population growth. Lynx hunting is generally conducted by large hunting teams who first locate lynx from their tracks in the snow along roads, which are then used to track lynx and eventually encircle and hunt them. On the other hand, poaching normally occurs opportunistically during the hunting season for other species (mainly in autumn) when snow is absent, which makes roads less important because tracks are not actively sought [33]. Due to the importance of roads for the pursuit of legal hunting, we would expect the availability of roads to have a critical effect on the exposure of animals to legal hunting, but not poaching. The occurrence of a marked, human-caused, spatial heterogeneity in habitat between and within individual home ranges on the one hand, and the strong impact of humans, which act as the main predator for lynx on the other hand, makes lynx a good model to study habitat selection in relation to fitness variation across spatial scales.

At the landscape scale, lynx tend to trade safety for food, by establishing in areas with a relatively high human accessibility, where their main prey, roe deer *Capreolus capreolus* [34], [35], generally occur [31], [36], but strongly avoid the most human-disturbed areas, where roe deer abundance is also higher [31]. The HHS hypothesis implies that animals should select against the most limiting factor at coarser scales to maximise their fitness, and if successful, they will select against the second most limiting factor at a finer scale. However, if they fail, the HHS hypothesis predicts that they will consistently select against the same limiting factor at finer scales until they succeed in avoiding characteristics associated with it, leading the influence of a limiting factor to persist over a broad range of scales (i.e. a broad domain, [37]). The HHS hypothesis thus allows the formulation of several predictions regarding the value of a limiting factor and its influence on habitat selection at multiple spatial scales. We thus expect human disturbance to impose a greater impact on lynx survival at large (home range) than at fine (habitat selection within home ranges) scales (**P1a**). At fine scales, once safety has been secured, lynx should not avoid human disturbance anymore, and the search for food should be favoured (**P1b**). However, as a limiting factor can span several spatial scales, we also expect hierarchical effects of human disturbance to occur on lynx mortality, with fine-scale selection of human disturbance having a stronger impact on mortality for lynx already established in high disturbance areas (**P2a**). As a consequence, for lynx that failed to secure their safety at large scales, we expect a compensatory response at a finer scale, so that individuals that established close to humans should avoid disturbance to a larger extent than individuals that established in remote areas. We thus expect a functional response in habitat preference [38]–[40] to occur in fine scale habitat selection (**P2b**).

## Materials and Methods

### Lynx monitoring

**Figure 1:**
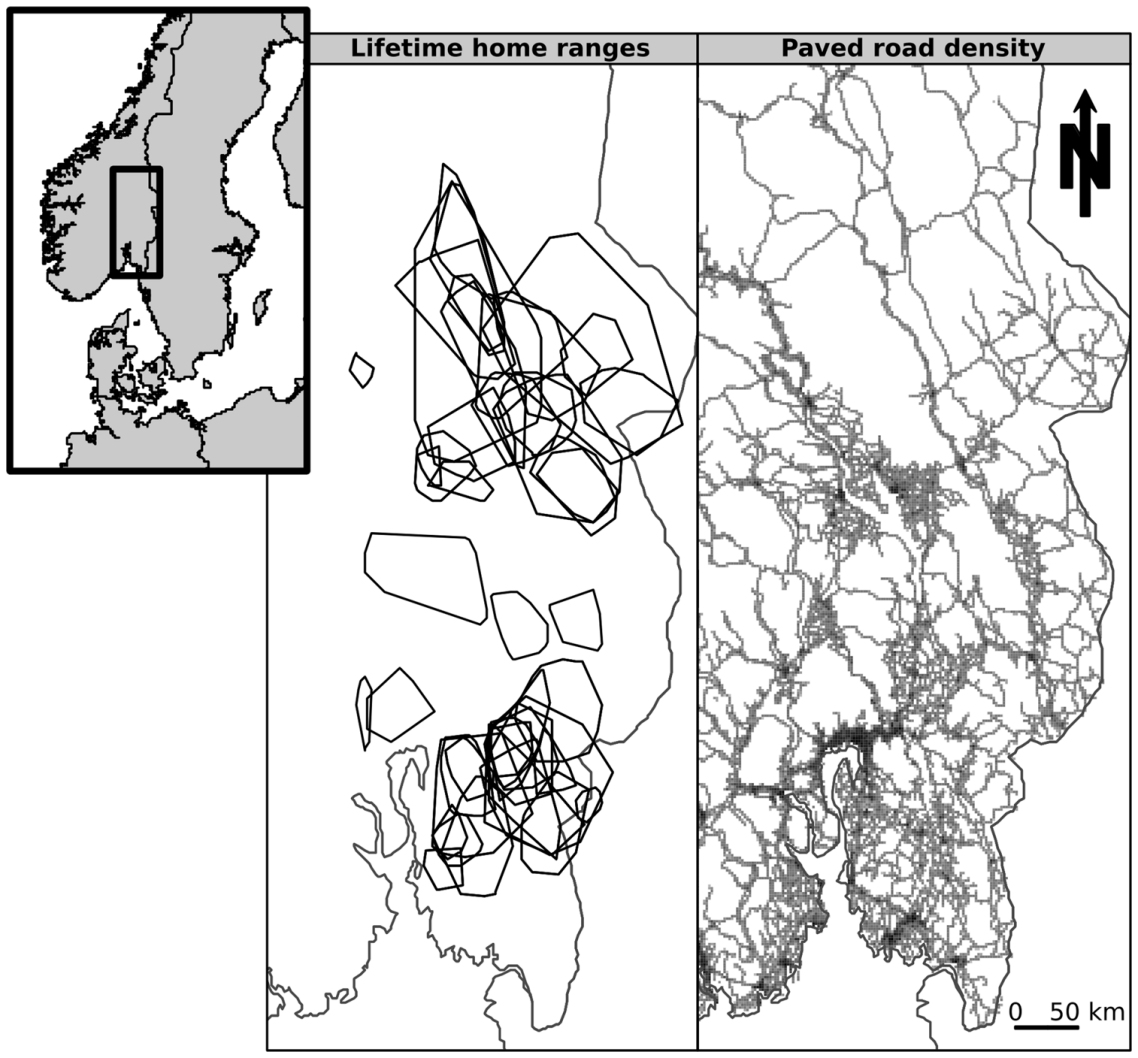
The study area in south-eastern Norway between ca. 59–62°N and 10–12°E. Lifetime home ranges are displayed on the left panel, while paved road density is represented in the right panel (light grey is low road density and dark grey is high road density).

Between 1995 and 2008, lynx were intensively monitored by telemetry (VHF or GPS collars) in two adjacent study sites in southern Norway (in the counties of Hedmark, Østfold, and Oslo & Akershus), between approximately 59–62°N and 10–12°E (Figure 1). We sexed each individual, and the age was either known exactly (lynx monitored from birth or retrospectively aged by sectioning a tooth following their death) or estimated *a minima* (estimated from length, weight, and dental characteristics of the lynx). After visual exploration of the telemetry locations, we excluded juvenile and dispersing individuals to retain only resident individuals (i.e. adults that were established in a home range after dispersal) in the analyses (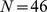). Based on the life cycle of lynx and the timing of the hunting season, we defined three biological seasons: the breeding season in May–August (most births occur around May 28^th^±5 days, 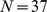, J. D. C. Linnell, Norwegian Institute for Nature Research, unpublished data); the game-hunting season in September–December, as the snow-free season in which most hunting activity for other species brings many hunters into the forest; and the lynx-hunting season in January–April, as the season with snow cover and legal lynx hunting (February 1^st^ to March 31^ st^).

Mortality events were detected during and after the telemetry monitoring until the lynx was found dead. Cause of death was attributed to legal hunting, poaching (either confirmed, probable, or possible), and other causes [33]. Poaching is by definition very difficult to quantify. Confirmed poaching included cases where the lynx carcass was found with a gunshot wound (outside the lynx hunting season), when the radio-transmitter was found at the bottom of a lake and the collar had been cut off from the lynx, or a collar was found smashed. To separate between probable poaching, possible poaching and unknown disappearance (e.g. transmitter failure) we used several criteria. Probable poaching included cases where the lynx had two separate transmitters (i.e. one radio-collar and one implanted radio-transmitter) and both of the transmitters suddenly disappeared, or when a female with kittens disappeared and the (collared) kittens were observed alone. It also included cases where a resident female with a new collar disappeared, and no family groups were snow-tracked in the area in subsequent seasons. Possible poaching included cases where a resident adult lynx suddenly disappeared and was not recovered despite intensive radio-tracking from the air immediately after the disappearance, in the absence of any evidence of technical problems (e.g. strange or weak signals) with the radio-transmitter. Otherwise, the lynx was classified as having an unknown fate.

### Ethics statement

Lynx were captured by hand at their natal lairs when less than 2 months old or in walk-through box-traps, spring-loaded foot-snares placed at kill sites, treed using trained dogs, or darted from a helicopter or from the ground. The capture methods were being constantly refined and fine-tuned to minimise the risk of injury or death to the animals [41]. In particular, the design and alarms of box traps and snares were modified to allow response time of less than 12 hours, and 20 minutes, respectively, and a safety net was used to catch animals treed with hounds. Juvenile and adult lynx were darted using a combination of medetomidine (Zalopine®) and ketamine (Narketan 10®), with lower doses for adults captured in box traps (calm animals) and juveniles. Kittens were captured by hand in their natal lairs, and weighed and immobilised with a combination of medetomidine (Domitor®) and ketamine (Ketalar®) intramuscularly. Animals were monitored during anaesthesia, which was reversed using atipamezole (Antisedan®) intramuscularly. Lynx were usually allowed to awaken without the presence of observers in order to minimise stress, but they were monitored remotely using telemetry techniques. Capture and handling protocols were approved by the Norwegian Experimental Animal Ethics Committee and followed their ethical requirements for research on wild animals. In addition, permits to capture wild animals were provided by the Norwegian Directorate for Nature Management (permit numbers 08/127430, 07/81885, 07/7883, 2004/48647, 201/01/641.5/FHB, 127/03/641.5/fhb, 1460/99/641.5/FBe, 1081/97/641.5/FBe and NINA 1/95). Permission to capture animals was always obtained from the land-owner, irrespective of if it was private or belonged to one of the state land management authorities. Additionally, permission was obtained from the Office of Environmental Affairs of the relevant county if capture was conducted within a protected area.

### Habitat characteristics

The study site represents a gradient of elevation from north to south, corresponding to a similar gradient in human use of the landscape. As home ranges occupied very large and variable areas in the study site (lifetime home range size was on average 839.50±123.84 [mean ± SE] km^2^), we described the landscape with a resolution of 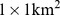 for every environmental layer (i.e. leading to an average of more than 800 pixels per lynx home range). We used the habitat typology based on the Global Land Cover 2000 database [42] to compute the proportion of forest and agricultural fields per square kilometre. Data on road density were obtained from the Norwegian Mapping Authority to define human accessibility. Paved and forest road densities were calculated as the total length of paved and forest roads (km) within each 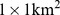 pixel, respectively. Paved roads are high-traffic roads, ranging from municipal roads to national highways; on the contrary, forest roads are usually private roads with little traffic, in connection to farming, logging or recreational resorts. In addition, human density throughout the area, measured as the number of inhabitants per square kilometre, completed the description of human disturbance [43].

As the telemetry monitoring varied in intensity throughout the study period we randomly selected one location per day when more than one daily location was recorded to avoid any sampling bias in the characterisation of home range composition and habitat use. We estimated lifetime home ranges using 95% minimum convex polygon [44] and determined their average composition for each environmental factor (Table 1) as a measure of second-order selection [28]. For each lynx, we also computed their use within the home range in each season, as the average characteristics recorded at each location for a given season. We used it to estimate seasonal habitat selection as a measure of third-order selection [28], i.e. the difference between what was available to each individual within their lifetime home range (corresponding to the average composition of the home range) and what was actually used during a given season [28], [45], for each environmental factor (Table 1). This measure is equivalent to the marginality [46], [47].

**Table 1:** Environmental covariates used in survival and habitat selection analyses.

Name	Description	Mean ± SE	*c_v_*
Fields	Proportion of fields per km^2^	0.08±0.92×10^−3^	223%
Forest	Proportion of forest per km^2^	0.62±1.80×10^−3^	57%
Human	Number of inhabitants per km^2^	39.00±1.53	765%
Paved	Total length of paved roads (km) per km^2^	0.46±6.20×10^−3^	262%
FRoads	Total length of forest roads (km) per km^2^	0.89±4.92×10^−3^	108%

All covariates were at the resolution of 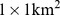. Land cover characteristics were derived from the Global Land Cover 2000 database; road densities, and human density, were obtained from the Norwegian Mapping Authority, and Statistics Norway, respectively. Means, standard errors (SE) and coefficients of variation (*c_v_*) are given in the study area.

We standardised all home range composition covariates, by subtracting their mean and dividing by their standard deviation, to improve model convergence and to ensure that all covariates were on the same scale to compare their respective importance. Third-order selection covariates, as defined above, were scaled by dividing them by their standard deviation, but were not centred, so that the sign of the seasonal habitat selection (either positive or negative for characteristics greater or less than the lifetime home range characteristics) remained unchanged.

### Statistical analyses

#### Assessing the hierarchy of limiting factors

To test the predictions associated to the hierarchy of limiting factors, we first fitted a set of semi-parametric proportional hazards (SPPH) models [48] on the follow-up time (expressed in number of seasons, i.e. 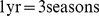) given the status of the lynx at the end of the monitoring period. All competing survival models were of the form:
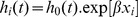
where the instantaneous risk of mortality of an individual 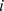 in a given season 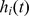 (i.e. the hazard rate) is modelled as a function of the baseline hazard experienced by all individuals (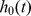) and a set of environmental factors (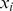, which represent the home range composition and seasonal habitat selection). In the semi-parametric approach, weak assumptions about the baseline hazard 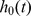 are made. The relative risk of death for individuals that differ with respect to a single covariate 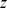 is given by the ratio between the hazards of two individuals 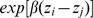, and is then independent of both the baseline hazard and time. The covariate effects (the 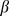
's) are then estimated using a partial likelihood that does not require estimating of 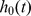.

As we are mostly interested by the influence of temporally varying risk factors (i.e. seasonally-varying selection) on lynx mortality, we used an age-based model, as advocated in [48]. With this approach, the influence of age is modelled non-parametrically, and the baseline hazard accounts for seasonal patterns that are consistent across years. On the other hand, temporally varying covariates are modelled parametrically as they may cause individuals of the same age (but born in different years) to have different mortality rates.

We proceeded in two steps: We first worked on the mortality caused by legal hunting, using only data collected during the lynx-hunting season. Secondly, we considered the mortality caused by poaching only, including probable and possible poaching (see above), in all seasons together, because poaching occurs all the year round. For both approaches, mortality events caused by other means than the one of interest (either legal hunting or poaching) were considered as censored observations, i.e. the individual is considered alive until the end of the season. Specifically, at each step, we contrasted three different models that corresponded to our predictions, by order of complexity: 1) a null model in which the relative hazard is not affected by environmental factors; 2) a model including environmental covariates at both second and third selection orders, i.e. the home range composition and marginality measurements (**P1a**); 3) a model including an interaction term for each environmental covariate between both scales to account for the aforementioned potential hierarchical effects (**P2a**). We also refined the later model by including only interaction terms for the covariates that are known to directly affect both lynx mortality and food availability, i.e. the density of paved and forest roads, and the proportion of fields [31], [36]. We then used an information theoretic framework to rank competing models based on 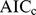 [49]; models within a 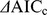 of 2 were considered the most plausible, with substantial empirical support. In addition, we used Akaike weights (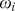) as the weight of evidence in favour of a model being the actual best model in the set of competing models [49].

#### Estimating habitat selection

The selection of specific habitat characteristics within the home range during a given season corresponds to a particular case of a discrete-choice model, referred to as a matched case-control study, with two units in the choice set [50], the current habitat use during a given season being consistently chosen and the lifetime home range composition defining what is available to the animal. In this context, the probability of selecting specific characteristics during a given season 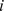 is:
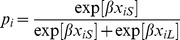
where 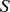 stands for the habitat use during a given season, while 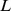 stands for the lifetime home range characteristics. Dividing all terms through by the second exponential term in the denominator produces:



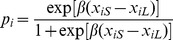



In this special case, the estimation of the resource selection function can thus be reduced to fitting a logistic regression function on the differences with no constant term. This is then equivalent to 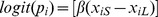, where 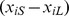 is a measure of third-order seasonal habitat selection, computed as the difference between home range composition and seasonal use. We can finally rewrite the last model as 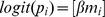, where 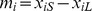 is the marginality (see above).

As for the survival analysis, we proceeded in two steps, using first data collected during the lynx-hunting season only, and second, data all the year round. Again, at each step, we contrasted three different models that correspond to our predictions: 1) a null model with no habitat selection; 2) a model of seasonal habitat selection including environmental factors at the third selection order (**P1b**); 3) a model of habitat selection differing according to resource availability, including interactions terms between the seasonal use and the lifetime home range composition for each environmental factor (**P2b**). Under this model, habitat selection at the individual level varies in response to changes in lifetime home range characteristics, which enabled us to test for a functional response in habitat preference (i.e. a change in third-order selection regarding varying availability at the second order [38]). Finally, as male lynx in Norway are generally found closer to human activity than females [51], we considered a fourth model of sex-specific fine-scale habitat selection (including females as the reference). As for the survival analyses (see above), we used an information theoretic framework to rank competing models based on 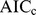 [49]. All analyses were conducted using R [52], with the help of the R packages “adehabitatHR” for the estimation of home ranges [53] and “MuMIn” for multi-model inference [54].

## Results

### Monitoring of radio-collared lynx

Forty-six individual lynx (25 females and 21 males) were monitored for an average of 7.81±1.04 (mean ± SE) seasons, which corresponds to 2.6 years, with an average of 171.61±20.04 (mean ± SE) locations per individual after subsampling one location per day (see *Material and Methods*). Technology has evolved throughout the study period, from VHF to GPS-based telemetry. For the purposes of this study, we assumed the accuracy of all locations to be 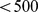 m. Among all individuals, 37 lynx (80.4%) were killed or found dead during the study period; 19 died from legal hunting, 5 from confirmed or probable poaching, 9 from possible poaching, and 4 from other causes. The final fate of 9 lynx could not be determined, and they were considered to have been alive until telemetry contact was lost or they dispersed from the study area. Altogether, the survival curve indicated a median lifespan of 15 seasons, i.e. exactly 5 years.

### Mortality from legal hunting and poaching

Eleven females and 8 males were killed from legal hunting. The most parsimonious model of mortality from legal hunting was the simple hierarchical model, which only included interaction terms between both scales for the selection of paved and forest road and agricultural fields (Table 2). This model received the highest empirical support (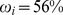) and was retained as the best model from the set, although the non-hierarchical model provided a similar fit (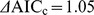) with, however, a lowest empirical support (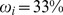). The full model that included all interaction terms and the null model only received limited support (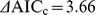 and 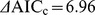, corresponding to 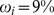 and 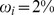, respectively, Table 2).

**Table 2:** Candidate models of lynx survival from legal hunting during the lynx-hunting season in Norway.

Model	*K*	*LL*	AIC	ΔAIC_C_	*ω_i_*
*Hier*	13	−27.29	83.6	0	0.56
*Env*	10	−31.43	84.6	1.05	0.33
*Full*	15	−26.61	87.2	3.66	0.09
*Null*	0	**−**45.28	90.6	6.96	0.02

All models were of the form 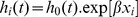, where the hazard rate 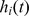 is modelled as a function of the baseline hazard 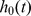 and a set of environmental factors 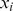: the *Env* model included environmental covariates at both large and fine scales, the *Hier* model included interaction terms on road densities and proportion of fields, the *Full* model included all interaction terms, and the *Null* model only estimated the intercept. The model selection followed an information-theoretic approach based on the number of parameters (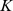), maximum log-likelihood (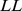), modified Akaike information criteria (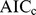), relative 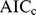 values (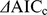), and 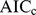 weight (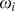) of each model.

**Figure 2:**
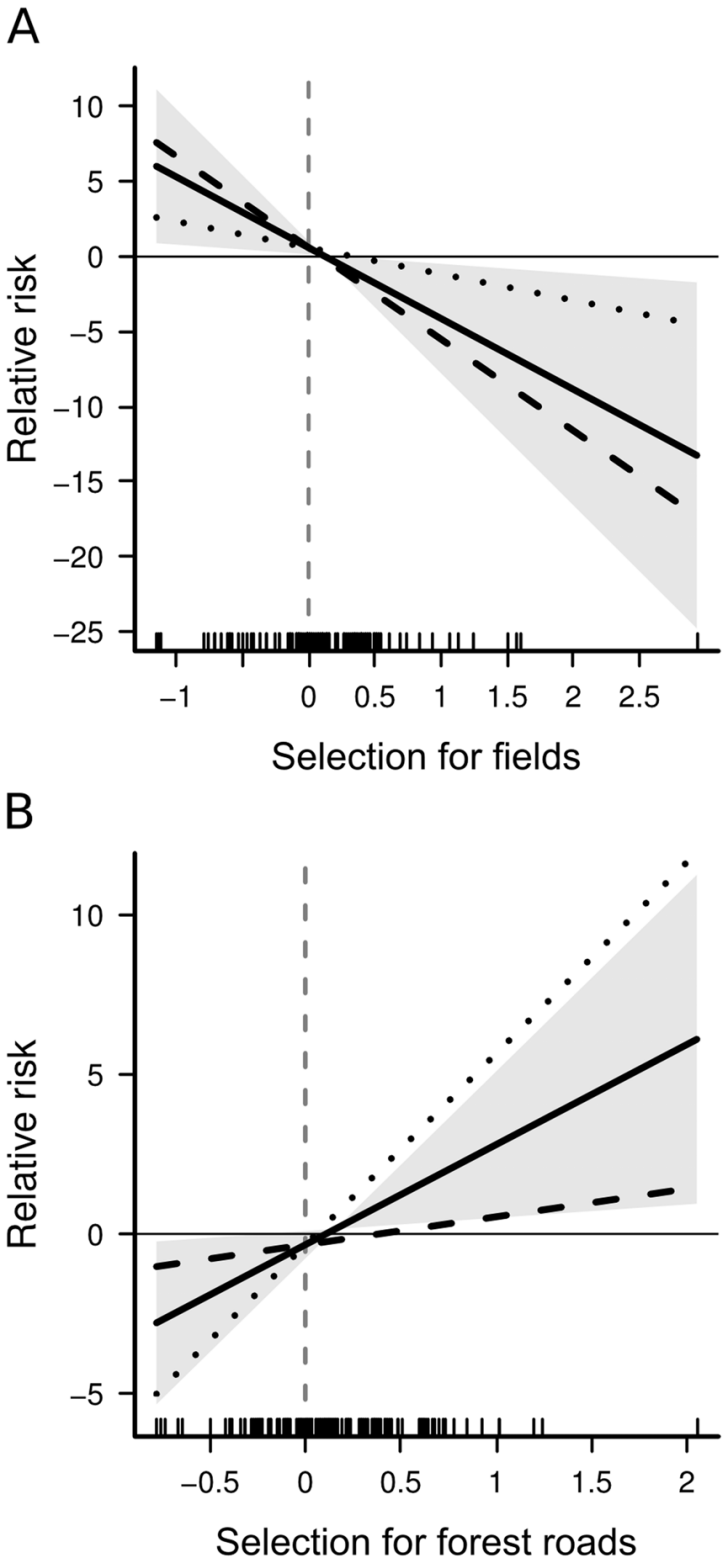
Relative risk of lynx being legally killed during the lynx-hunting season in Norway regarding the proportion of fields (A), and the forest road density (B). The relative risk is displayed on a log scale (i.e. on the scale of the linear predictors), as a function of third-order selection (X-axis) and for different levels of second-order selection: The solid, dashed, and dotted lines, correspond to the mean and the 5% and 95% percentiles of the home range composition for each variable (i.e. 8.7%, 0.9% and 25.5% for the proportion of fields, and 0.963, 0.678 and 1.323 km of forest road per km^2^, respectively). To improve clarity, the 95% confidence interval is only provided for the mean response. Each observation is indicated by a vertical segment on the X-axis.

The best model of legal-hunting mortality did not violate the proportional hazard assumption for any variable (all 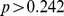). The risk of being legally killed during the lynx-hunting season decreased with forest road density in the home range (Table 3). Lynx selecting for a higher proportion of fields within their home range had a lower risk of being legally killed, but this decreasing effect on the risk almost vanished at high proportions of fields in the home range, as indicated by the positive interaction between the second-order and third-order habitat selection for this variable (Table 3, Figure 2A). Conversely, the selection within home ranges of higher paved and forest road densities strongly increased the risk of being legally killed during the lynx-hunting season, the later being reinforced by a positive interaction with forest road density in the home range (Table 3, Figure 2B). Other covariates had confidence intervals largely overlapping with zero (Table 3).

**Table 3:** Results of the hierarchical model of lynx survival from legal-hunting mortality during the lynx-hunting season in Norway.

Covariate	*β*		2.5% CI		97.5% CI
*Home range composition (2^nd^ order)*					
FieldsLHR		0.63		**−**1.30	2.56
ForestLHR		**−2.12**		**−3.93**	**−0.31**
HumanLHR		**−**3.01		**−**7.80	1.79
PavedLHR		0.78		**−**3.19	4.74
FRoadsLHR		1.31		**−**0.06	2.67
*Habitat selection (3^ rd^ order)*					
FieldsHS	**−2.33**		**−4.35**		**−0.31**
ForestHS	**−**0.32		**−**1.08		0.43
HumanHS	1.15		**−**0.61		2.92
PavedHS	**1.98**		**0.60**		**3.36**
FRoadsHS	**1.56**		**−0.06**		**2.67**
*Interaction terms (2^ nd^ ×3^ rd^ orders)*					
FieldsLHR: FieldsHS	**0.73**		**0.01**		**1.46**
PavedLHR: PavedHS	0.26		**−**0.97		1.49
FRoadsLHR: FRoadsHS	**0.81**		**−0.07**		**1.70**

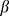
: regression coefficients, 2.5% CI and 97.5% CI: confidence intervals computed at the 95% interval. For the variable names, “LHR” stands for lifetime home range composition (i.e. 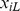), “HS” stands for seasonal habitat selection (i.e. 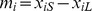). Coefficients with 90% CI non-overlapping with zero are highlighted in bold.

Consequently, the proportion of forest was the only limiting factor at the home range scale, and was superseded by human-related factors within the home range, namely the proportion of fields and the paved and forest road densities. Hierarchical effects arose for the selection of higher proportions of fields (with the effect of third-order selection being cancelled by the second-order selection) and higher forest road density (with the effect of third-order selection being reinforced by the second-order selection).

Poaching occurred throughout the year (lynx-hunting season: 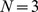, breeding season: 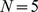, game-hunting season: 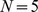), and impacted almost equally females (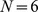) and males (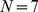), which does not justify the inclusion of a season or sex effect. The most parsimonious model was the null model with an empirical support of 96%, and the second best model in the set was the hierarchical model (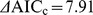). As a consequence, we could not detect any influence of environmental variables at either scale on the annual risk of being poached. The null model indicated a median life expectancy regarding poaching of 21 seasons ( = 7 years).

### Habitat selection during the lynx-hunting season

The most parsimonious model of habitat selection during the lynx-hunting season was the hierarchical model, which included both environmental factors at the third selection order, and their interactions with lifetime home range composition (Table 4). The best alternative models performed poorly in comparison, with a 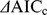 of 27.08 for the model without the interaction terms, and 29.61 for the model including sex. The null model had a 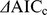 of 77.71. These high 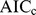 differences accounted for a complete empirical support (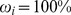) for the hierarchical model (Table 4).

**Table 4:** Candidate models of lynx third-order habitat selection in Norway during the lynx-hunting season, and all the year round.

	Lynx-hunting season					All the year round				
Mo del	*K*	*LL*	AIC_C_	ΔAIC_C_	W_i_	*K*	*LL*	AIC_C_	DAIC_C_	W_i_
*Hier*	10	**−**43.83	109.4	0	1.00	10	**−**134.1	288.9	0	1.00
*Env*	5	**−**63.02	136.5	27.08	0.00	5	**−**153.6	317.3	28.40	0.00
*Sex*	10	**−**58.63	139.0	29.61	0.00	10	**−**150.2	321.1	32.22	0.00
*Nul*	0	**−**93.58	187.1	77.71	0.00	0	**−**240.5	481.0	192.15	0.00

All models were of the form 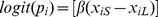, where the probability of selecting the actual use during a given season 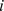 is modelled as a function of a set of environmental factors measured for the lifetime home range composition (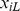) and seasonal use (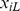): the *Hier* model included all interaction terms between both scales, the *Env* model included only the third-order selection measurements, the *Sex* model included an effect of sex in interaction with third-order selection measurements, and the *Null* model only estimated the intercept. The model selection followed an information-theoretic approach based on the number of parameters (*K*), maximum log-likelihood (*LL*), modified Akaike information criteria (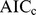), relative 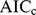 values (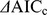), and 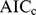 weight (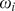) of each model.

**Figure 3:**
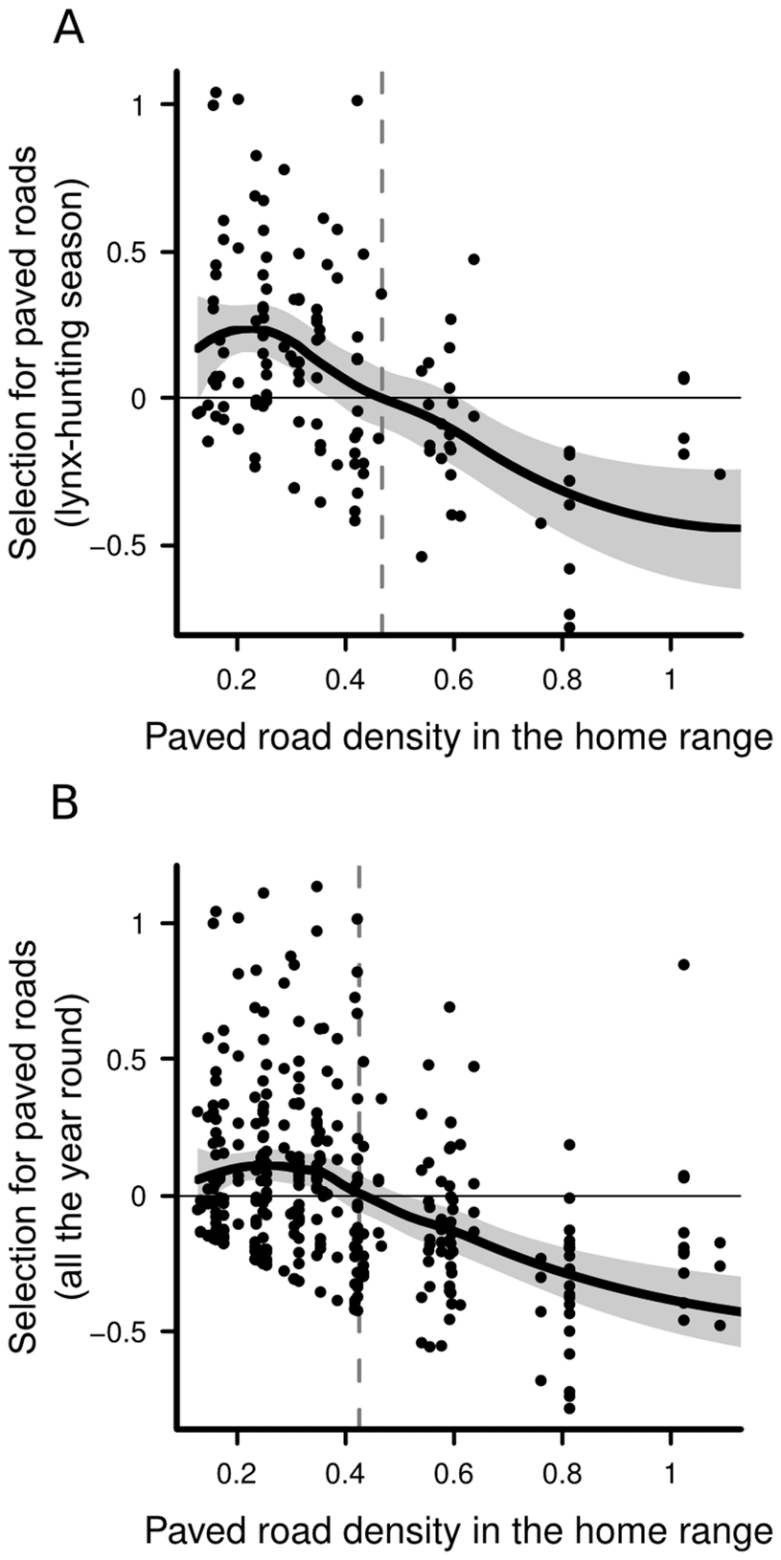
Lynx habitat selection regarding paved road density in Norway during the lynx-hunting season (A), and all the year round (B). The seasonal selection for paved roads (i.e. 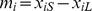) is modelled as a function of the paved road density in the lifetime home range (i.e. 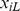). For better readability, one individual established at very high road density (2.16 km/km^2^) was removed from both plots, but not from the analyses. A local polynomial regression, with 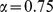 and 2 degrees, was fitted to the data (bold line with 95% confidence interval in grey) to illustrate the output of the hierarchical habitat selection model. A dashed line indicates where the switch from a positive to a negative selection (i.e. avoidance) occurs (at 0.47 km/km^2^ during the lynx-hunting season, and 0.41 km/km^2^ all the year round).

The hierarchical model had variance inflation factors (VIF) consistently 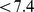, indicating no marked multicollinearity [55]. According to the hierarchical model, lynx selected a high proportion of fields and forests within their home ranges (Table 5). This selection was, however, modulated by the overall availability of fields and forests in their lifetime home ranges: the selection for fields decreased as their availability increased in the lifetime home range, while the selection for forests increased as their availability increased in the lifetime home range. Conversely, although the selection of paved road density was generally not different from zero, there was a strong negative interaction between their selection and their availability in the lifetime home range (Table 5). Lynx established in areas below a threshold of 0.47 km/km^2^ of paved roads selected areas with higher densities of paved road within their home range, after which they consistently avoided paved roads within their home range (Table 5, Figure 3A). Other covariates had confidence intervals largely overlapping with zero (Table 5).

**Table 5:** Results of the hierarchical models of lynx habitat selection in Norway during the lynx-hunting season, and all the year round.

	Lynx-hunting season			All the year round		
Covariate	*β*	2.5% CI	97.5% CI	*β*	2.5% CI	97.5% CI
*Habitat selection (3^rd^ order)*						
FieldsHS	**2.53**	**1.22**	**4.12**	**1.38**	**0.79**	**2.03**
ForestHS	**2.44**	**1.55**	**3.55**	**2.32**	**1.80**	**2.90**
HumanHS	0.31	**−**0.79	1.82	**−**0.21	**−**0.85	0.34
PavedHS	**−**0.45	**−**1.45	0.51	**−**0.05	**−**0.50	0.41
FRoadsHS	0.53	**−**0.31	1.46	**0.73**	**0.29**	**1.23**
*Interaction terms (2^ nd^ ×3^ rd^ orders)*						
FieldsLHR: FieldsHS	**−0.63**	**−1.34**	**−0.01**	**−0.32**	**−0.68**	**0.05**
ForestLHR: ForestHS	**1.19**	**0.51**	**1.91**	**0.82**	**0.39**	**1.25**
HumanLHR: HumanHS	**−**0.18	**−**0.58	0.31	0.04	**−**0.18	0.38
PavedLHR: PavedHS	**−2.79**	**−4.67**	**−1.41**	**−1.25**	**−1.95**	**−0.65**
FRoadsLHR: FRoadsHS	0.33	**−**0.36	0.985	**−**0.20	**−**0.72	0.24

*β*: regression coefficients, 2.5% CI and 97.5% CI: confidence intervals computed at the 95% interval. For the variable names, “LHR” stands for lifetime home range composition (i.e. 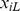), “HS” stands for seasonal habitat selection (i.e. 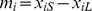). Coefficients with 90% CI non-overlapping with zero are highlighted in bold.

### Year-round habitat selection

As for the lynx-hunting season, the most parsimonious model of year-round habitat selection was the hierarchical model, which included both environmental factors at the third selection order, and their interactions with lifetime home range composition (Table 4). The best alternative models had a 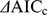 of 28.40 for the model without the interaction terms, and 32.22 for the model including sex. The null model had a 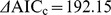. These high 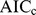 differences accounted for a complete empirical support (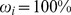) for the hierarchical model (Table 4).

The hierarchical model had variance inflation factors (VIF) consistently 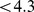, indicating no marked multicollinearity [55]. Year-round habitat selection was fairly similar to the selection during the lynx-hunting season: lynx selected a high proportion of fields and forests within their home ranges (Table 5), but this selection was modulated by the overall availability of fields and forests in their lifetime home ranges. The selection for fields decreased as their availability increased in the lifetime home range, while the selection for forests increased as their availability increased in the lifetime home range. Conversely, although the selection of paved road density was generally not different from zero, there was a strong negative interaction between their selection and their general availability in the lifetime home range (Table 5). Lynx established in areas below a threshold of 0.41 km/km^2^ of paved roads selected for areas with higher densities of paved roads within their home range, after which they consistently avoided paved roads within their home range (Table 5, Figure 3). The only difference with habitat selection during the lynx-hunting season occurred with respect to the selection of forest road density, which was positive all the year round. Other covariates had confidence intervals largely overlapping with zero (Table 5).

## Discussion

Several authors have emphasised the need to concurrently study habitat and individual performance to identify their relationships at multiple spatial scales to better understand spatial variation in population dynamics [56]–[58]. Using human disturbance as a driver of heterogeneity in individual fitness, this study successfully related mortality (a direct fitness component), home range characteristics, and habitat selection in a common framework [4], providing a mechanistic explanation of the risk of mortality based on animal behaviour. We were able to formally test the hierarchy of limiting factors across spatial scales, demonstrating support for the Hierarchical Habitat Selection (HHS) hypothesis. While we were unable to detect a stronger impact of humans at large (i.e. characteristics of the home range) than at fine (i.e. selection within home ranges) scales (**P1a** rejected), we demonstrated hierarchical effects in the impact of human disturbance, where a high level of disturbance at the large scale reinforced its impact at the fine scale (**P2a** supported). Conversely, we demonstrated that lynx avoided areas with only the highest road density within their home ranges all the year round, thus supporting compensatory habitat selection (**P1b** and **P2b** supported).

The theory of limiting factors is strongly grounded in hierarchy theory, in which processes occurring at larger scales constrain lower-level processes in a nested fashion, enabling the avoidance of the most limiting factors at large scales [7]. At a large spatial scale, lynx traded foraging, measured through an index of roe deer abundance, for safety, indexed by measures of human access and disturbance [31]. The results from this study indicate that at a fine spatial scale, the consequences of this trade-off apparently constrain lynx that establish in areas highly accessible to humans to compensate with a strong avoidance of highly disturbed areas within their home ranges. This compensation suggests that lynx make “the best of a bad situation” – while the distribution of the population might lead lynx to establish their home ranges in riskier areas, their large home ranges [29] allow them to secure their space use by avoiding areas associated with their main risk of death. Moreover, the selection for agricultural fields within the home range decreased the relative risk of mortality when the home range had low proportions of fields. Interestingly, lynx that had home ranges with a low proportion of fields displayed a clear selection pattern for field that vanished for lynx established in home ranges with more fields. Altogether, our study thus provides a clear demonstration of the hierarchical nature of habitat selection of lynx, with large-scale characteristics constraining fine-scale behaviours.

Our study, using an expanded data set (more animals) from 2 of the 4 study areas included in [33], revealed that 89% of documented deaths were associated with legal hunting and poaching, making human-caused mortality a critical factor for lynx fitness [32]. Nevertheless, lynx seemed to give priority to food at the expense of a greater risk, and only avoided the most human-disturbed areas, at both large and fine scales. It should nevertheless be noted that the statistical power associated with our survival analyses was relatively limited, especially for the risk of poaching (13 deaths from poaching vs. 19 deaths from legal hunting), which could have prevented us from identifying other limiting factors such as human impact at large scales. As previously reported in other systems [10], [11], our case study of lynx demonstrates that the most limiting factor for fitness does not consistently occur at the broader spatial scale. This can probably be explained by the conditions experienced by lynx in southern Norway, which live in areas characterised with low roe deer densities compared to those in continental Europe. In spite of densities as low as 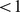 deer per km^2^ [59], lynx in southern Norway still specialise on roe deer, which are the most common prey species for lynx, especially during the lynx-hunting season when roe deer contribute to 83% of the biomass consumed by lynx [34]. In particular, poor food conditions, rather than predation risk, can become the most limiting factor at large spatial scale, and drive the avoidance of predation at finer scale, as shown for several ungulates in northern or Alpine areas or during winter [60]–[62].

Our findings regarding the hierarchical nature of selection across spatial scales imply the need for two modifications to the top-down hierarchical selection theory [9]. First, for a selection pattern to emerge, the environment should express some degree of heterogeneity at the focal scale [63]. In a completely homogeneous environment, there is nothing to select at all. Although fairly trivial, it has been mostly ignored in habitat selection studies (see, however, [10], [11]). Second, heterogeneity at a given scale alone cannot explain strong and seemingly contradictory selection patterns across spatial scales. A possible explanation is that, in relation to their variability across spatial scales, animals are able to mitigate the impact of the most limiting factors by the means of trade-offs across spatial scales [16], [64]. This situation should arise when the effects of multiple factors, such as predation risk and food limitation, occur at the same spatial scales [18]. Specifically, we suggest that the scale of potential limiting factors should be considered in agreement with the scale of their impact on the focal species, by assessing their consequences on individual fitness at different scales in relation to their heterogeneity [65], [66]. In our case, lynx have to trade security for food at large scales because of the relatively well-developed road network (1.58 km/km^2^ on average in the area) compared to their large spatial requirements (home ranges up to 3,000 km^2^ for males). In other words, given the low roe deer density, there is no possibility for lynx to establish their home ranges in entirely secure areas (i.e. without roads) if they are to eat, and lynx only avoid areas with the greatest level of road density. Consequently, lynx are forced to adjust their space use at a fine spatial scale too, by trading again their security for food, until the risk gets too high.

Our study identified a clear cost of human accessibility on lynx survival, which could lead to maladaptive behavioural responses [67], as can be the case in habitats modified by human activity or when the mortality is mostly caused by human harvesting [2], [68]. It is noteworthy that lynx affinity for areas with a high road density (i.e. high-risk areas) decreased as the road density increased, until being avoided at the highest densities. Lynx thus seem to correctly assess the source of disturbance, contrary to what is expected from the ecological-trap hypothesis [69], which expects that animals may fail to correctly assess habitat quality [2], [70]. Moreover, potential benefits associated with areas with a high road density might offset the cost of human accessibility on survival. Contrary to other predators such as grey wolves, which use roads to patrol their territories more efficiently [71], lynx likely select areas where their prey tend to be, and not roads *per se*. In southern Norway, areas with a high density of roads are generally low-lying areas close to fields and houses, where their main prey, roe deer, occur at high abundance due to the availability of high-quality forage and cover in close proximity [31], [36]. As a consequence, selection of areas with high mortality risks could be the result of a trade-off between survival and reproduction, although the latter remains to be evaluated before concluding about potential sinks in the system and the maladaptive nature of lynx habitat selection in human-dominated landscapes [3].

In the European multi-use landscape where large carnivore conservation occurs, human-caused mortality is directly related to human infrastructural development and access. In particular, while most large carnivores are able to cross roads [72], [73], road-related mortality can strongly impact population dynamics of large carnivores through collisions [74], [75], modification of animal behaviour [76], and increased accessibility of areas to hunters (reviewed in [77], [78]). Although vehicle collisions are only a minor issue for lynx in southern Norway, individuals that selected areas highly accessible to humans within their home range – i.e. with a high road density – were exposed to a high risk of mortality through hunting. Lynx responded to that threat by consistently avoiding the areas with the greatest hunting access. Many empirical studies have previously reported negative impacts of roads on wildlife, either with the direct effect of an increased hunting pressure [79], or the indirect effect of noise disturbance [80] or human activity on roads and in their vicinity [81]. Since our index of road density is only a combined proxy for all these factors, the mechanism involved in lynx avoidance of roads remains to be demonstrated. On the other hand, the different results for legal hunting and poaching accurately reflect our understanding of the way these different activities are conducted. The study area has a very high density of roads – mainly forest roads that penetrate to most areas – and is almost entirely private land. Lynx hunting is greatly facilitated by this network as these roads are driven in search of tracks, which are then the starting point for tracking lynx and eventual encirclement or drive hunt. For this to succeed it is crucial that the tracks are fresh; as lynx can move up to 40 km in a night, having many roads permits the tracks to be cut so that the encirclement only begins on the very freshest tracks. It is therefore very logical that roads relate to mortality risk from legal hunting. The crucial aspect here is that, although formally lynx hunting is tied to landownership, a tradition has developed where large hunting teams secure the permission of very many landowners to hunt lynx so that the hunting teams can follow the lynx across large areas wherever they go. This is not the case with other forms of hunting, such as moose, roe deer and small game, which are strictly tied to landownership as it represents a major source of revenue for landowners (meat and license sales) as well as an important part of landowners' recreational activity and culture. Hunters therefore penetrate all areas and are pretty much distributed across the whole landscape independent of road density. No areas are so remote from a road that they are difficult to access by hunters. At this time of year, lynx poaching cannot be facilitated by snow tracking so a poaching opportunity will only come if a lynx is visually seen. Therefore, it is not expected that roads will markedly influence hunter density, which in turn implies it should not influence poaching risk.

We demonstrated that lynx manage to live in quite heavily human-dominated areas, characterised by high mortality risks, by making behavioural decisions favouring their survival under such conditions. By showing that lynx, as many large carnivores [82], can persist in areas heavily dominated by human infrastructure, our results are important to assist planning for lynx recovery over large spatial scales. This was made possible by the collection of a large amount of individually based data, which is a prerequisite to understand population dynamics in relation to habitat selection [83]. The identification of different attributes of areas where lynx are at risk from legal harvest is a further step in being able to build spatially explicit models that link population viability to landscape, which are potentially powerful tools in conservation planning [84]. We found that poaching, while less intense than hunting, is less predictable so that lynx cannot mount any behavioural response to this threat. Poaching thus provides an uncontrolled and unpredictable source of additive mortality that makes it hard to develop a robust management system for lynx populations [85]. Finally, we highlighted the key importance of defining environmental heterogeneity at several spatial scales, which can have a tremendous impact on predator-prey relationships [26].
